# MED1 mediates androgen receptor splice variant induced gene expression in the absence of ligand

**DOI:** 10.18632/oncotarget.2672

**Published:** 2014-12-03

**Authors:** Gang Liu, Cynthia Sprenger, Pin-Jou Wu, Shihua Sun, Takuma Uo, Kathleen Haugk, Kathryn Soriano Epilepsia, Stephen Plymate

**Affiliations:** ^1^ Department of Medicine, University of Washington, Seattle 98104, WA; ^2^ Veteran Affairs Puget Sound Health Care System, Seattle 98104, WA

## Abstract

The appearance of constitutively active androgen receptor splice variants (AR-Vs) has been proposed as one of the causes of castration-resistant prostate cancer (CRPC). However, the underlying mechanism of AR-Vs in CRPC transcriptional regulation has not been defined. A distinct transcriptome enriched with cell cycle genes, e.g. UBE2C, has been associated with AR-Vs, which indicates the possibility of an altered transcriptional mechanism when compared to full-length wild-type AR (AR^fl^). Importantly, a recent study reported the critical role of p-MED1 in enhancing UBE2C expression through a locus looping pattern, which only occurs in CRPC but not in androgen-dependent prostate cancer (ADPC). To investigate the potential correlation between AR-V and MED1, in the present study we performed protein co-immunoprecipitation, chromatin immunoprecipitation, and cell proliferation assays and found that MED1 is necessary for AR^v567es^ induced UBE2C up-regulation and subsequent prostate cancer cell growth. Furthermore, p-MED1 is bound to AR^v567es^ independent of full-length AR; p-MED1 has higher recruitment to UBE2C promoter and enhancer regions in the presence of AR^v567es^. Our data indicate that p-MED1 serves as a key mediator in AR^v567es^ induced gene expression and suggests a mechanism by which AR-Vs promote the development and progression of CRPC.

## INTRODUCTION

Castration-resistant prostate cancer (CRPC) occurs when androgen ablation therapy fails. Patients with CRPC have an average survival time of 16 to 18 months from identification of recurrence [1–3]. A variety of mechanisms have been proposed for progression that bypasses current therapies targeting the androgen receptor (AR), including production of intratumoral androgens, increased conversion of adrenal androgen to testosterone, and increased AR expression after hormone deprivation [4–7]. In addition, various cytokines and growth factors have been shown to activate AR through direct binding or by cross-talk mechanisms [8, 9]. Functionally, all of these mechanisms rely on continued activation of the AR through its ligand-binding domain (LBD).

However, the recent identification of androgen receptor splices variants (AR-Vs) provides an alternative explanation for the development of CRPC. AR-Vs have been identified by several independent groups in human prostate cancer cell lines, xenografts, metastases, and circulating tumor cells [10–15]. Most characteristically, these variants are devoid of the ligand binding domain (LBD) but retain the capability to engage transcriptional machinery and promote the regulation of AR-target genes. The potential role of AR-Vs in driving prostate cancer progression is supported by several independent correlative clinical studies describing the significant association of AR-Vs with advanced disease progression and a shorter survival period [15–18]. Among the constitutively active AR-Vs, AR-V7 (or AR3) and AR^v567es^ are the two most commonly described in advanced disease [17, 19]. AR-V7 has been reported in many prostate tissues both benign and malignant, while AR^v567es^ has only been seen in malignant prostate glands [10, 14, 18, 19]. Furthermore, the *AR3/V7* and *AR^v567es^* transgenic mouse models demonstrated that expression of AR variant in mouse prostate induced high-grade prostatic intraepithelial neoplasia (PIN) [20] and/or invasive prostatic carcinoma [21].

The mechanism of AR-Vs in CRPC transcriptional regulation still remains unclear. Present evidence suggests, in addition to activation of the classical AR target genes, constitutively active AR splice variants are associated with a distinct transcription program in prostate cancer cells as well as in prostate cancer xenografts displaying treatment-induced AR-Vs expression [22]. Importantly, this distinct expression signature is enriched with cell cycle genes compared to the canonical AR-ligand dependent gene signature. Very interestingly, another study also described a similar transcription program comprised of upregulated cell-cycle genes in the androgen-independent prostate cancer (AIPC) cell line LNCaP-abl [23]. Although the latter research did not involve the role of AR variant, the ubiquitin-conjugating enzyme E2C (UBE2C) was the most up-regulated cell cycle gene in both studies. UBE2C is an anaphase-promoting complex/cyclosome (APC/C) E2 ubiquitin-conjugating enzyme. It can inactivate the M-phase check point and enhance cell growth. UBE2C has been shown to be a prominent oncogene in solid tumors, and it is found overexpressed in various types of solid tumors including late-stage prostate cancer [24–27]. Taken together, these studies indicate the presence of a distinct gene expression profile in CRPC that is different from the canonical AR-dependent transcriptome, one that might be associated with different transcriptional machinery of AR-Vs. The underlying mechanism on how AR-Vs induce a distinct transcriptional profile remains to be elucidated.

Modulation of androgen receptor (AR) co-regulators might play a pivotal role in CRPC [28, 29]. Previous studies have indicated that epigenetic markers and collaborating transcription factors can be ascribed to androgen-independent prostate cancer, including histone markers, [30] FoxA1 [31, 32], MED1(Mediator complex subunit 1) [33] and FOXO1. [34] Chen et al showed p-MED1 could drive CRPC cancer growth through a looping pattern on the UBE2C locus [35]. The chromatin looping functional pattern occurred uniquely in CRPC but not in androgen-dependent prostate cancer (ADPC). Given the critical role identified for MED1 in mediating UBE2C expression in CRPC, we asked whether co-activator MED1 could interact with AR-Vs to mediate the downstream events of transcriptional regulation.

## RESULTS

### MED1 is necessary for AR^v567es^ induced UBE2C regulation and subsequent prostate cancer cell growth

**Figure 1:**
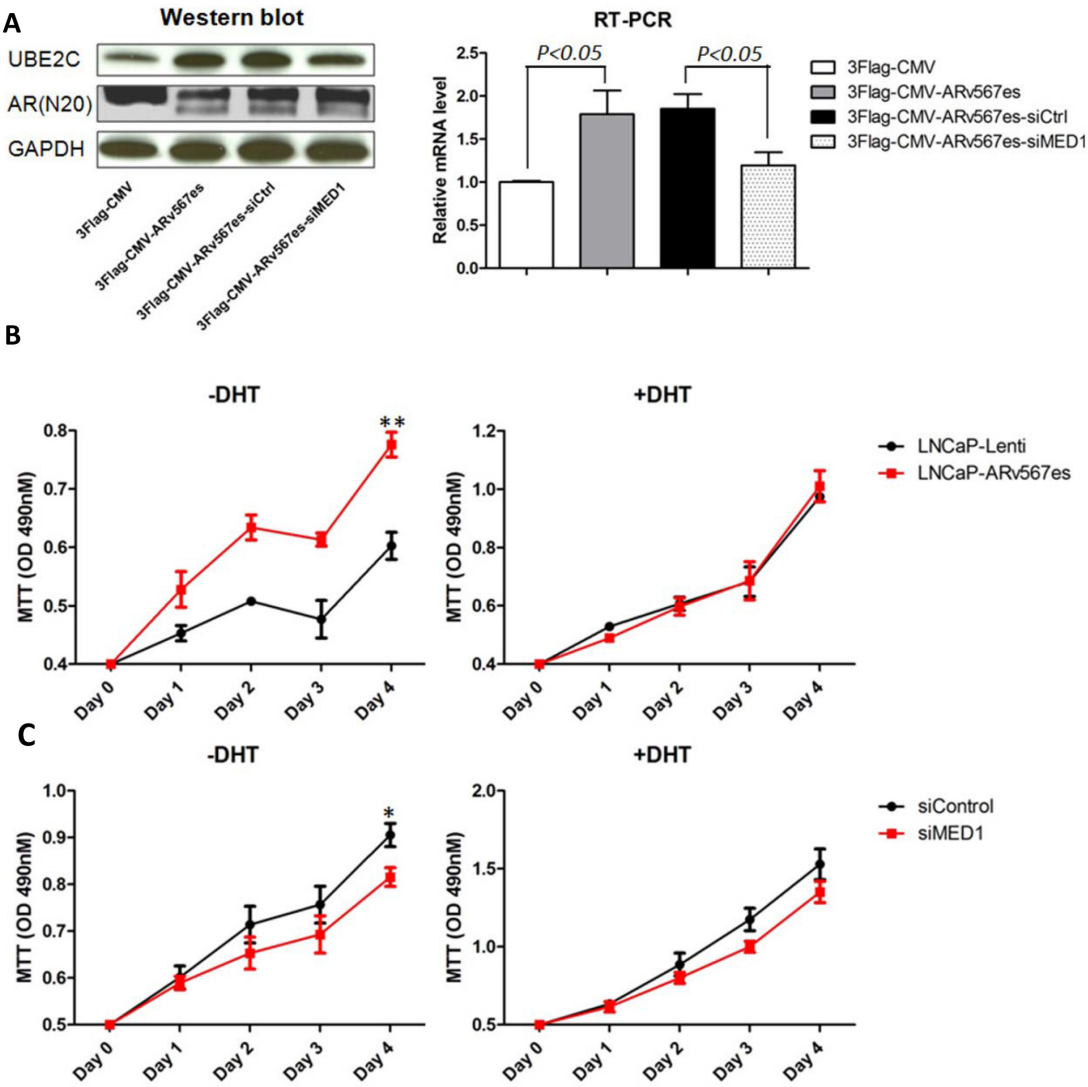
AR^v567es^ increased UBE2C expression and improved prostate cancer cell proliferation, which was blocked by MED1 silencing **(A)** Western blot and qRT-PCR showed significantly increased UBE2C expression in transiently transfected LNCaP cells with 3Flag-CMV-AR^v567es^ expression vector. Up-regulation of UBE2C by AR^v567es^ could be attenuated by MED1 siRNA at mRNA and protein level. **(B)** AR^v567es^ stable expressing cell line, LNCaP-AR^v567es^ showed significant higher proliferation rate (**p < 0.01) compared with Lenti virus empty vector control cell line in the absence, but not presence of DHT. **(C)** AR^v567es^ induced LNCaP cell proliferation could be blocked by MED1 silencing (*p < 0.05) when DHT was not present, but not significant with DHT.

We first confirmed the regulatory effect of AR^v567es^ variant on UBE2C expression in the AR-dependent cell line LNCaP. After performing transient transfection of an AR^v567es^ expression vector 3Flag-CMV-AR^v567es^, we assayed UBE2C protein and mRNA levels by Western blot and quantitative RT-PCR analysis. As shown in Figure 1A, the expression of UBE2C increased in the LNCaP-AR^v567es^ cells grown in media without DHT, consistent with previous reports [22]. To investigate whether MED1 is involved in this regulatory activity, MED1 expression was silenced by RNAi (Fig. S1). Subsequently, the up-regulation of UBE2C was significantly impaired at both the mRNA and protein level (Figure 1A). These data suggest MED1 is involved in AR^v567es^ mediated UBE2C expression.

The expression of AR^v567es^ in metastases portends a rapid progression of the tumor [18], which was also suggested by our *in vitro* study. The AR^v567es^ expressing stable cell line LNCaP-AR^v567es^ (see methods) grows faster than control LNCaP-Lenti cells in the absence, but not in the presence of DHT (Fig. 1B). To investigate whether there is a functional interaction between MED1 and AR^v567es^, we tested the effect of MED1 silencing on AR^v567es^ induced cell proliferation. Silencing of MED1 decreased proliferation of LNCaP-AR^v567es^ cells compared with scramble control (Fig. 1C and S2). This effect was more significant in the androgen-deprived condition. This phenomenon indicates MED1 is more involved with AR^v567es^ transcriptional regulation when the ligand is absent, but it is not actively interacting with full-length AR (AR^fl^) when androgen is present. QPCR was further performed to investigate the UBE2C expression in the stable cell lines (Fig. S3). Higher UBE2C expression was seen in the LNCaP-AR^v567es^ cell line in the absence of DHT; both DHT and siMED1 significantly inhibited UBE2C expression. Collectively, these data suggest MED1 plays an essential role in the AR^v567es^ induction of UBE2C and subsequent prostate cancer cell growth in an androgen-independent manner.

### MED1 is recruited to AR^v567es^ independent of full-length AR

**Figure 2:**
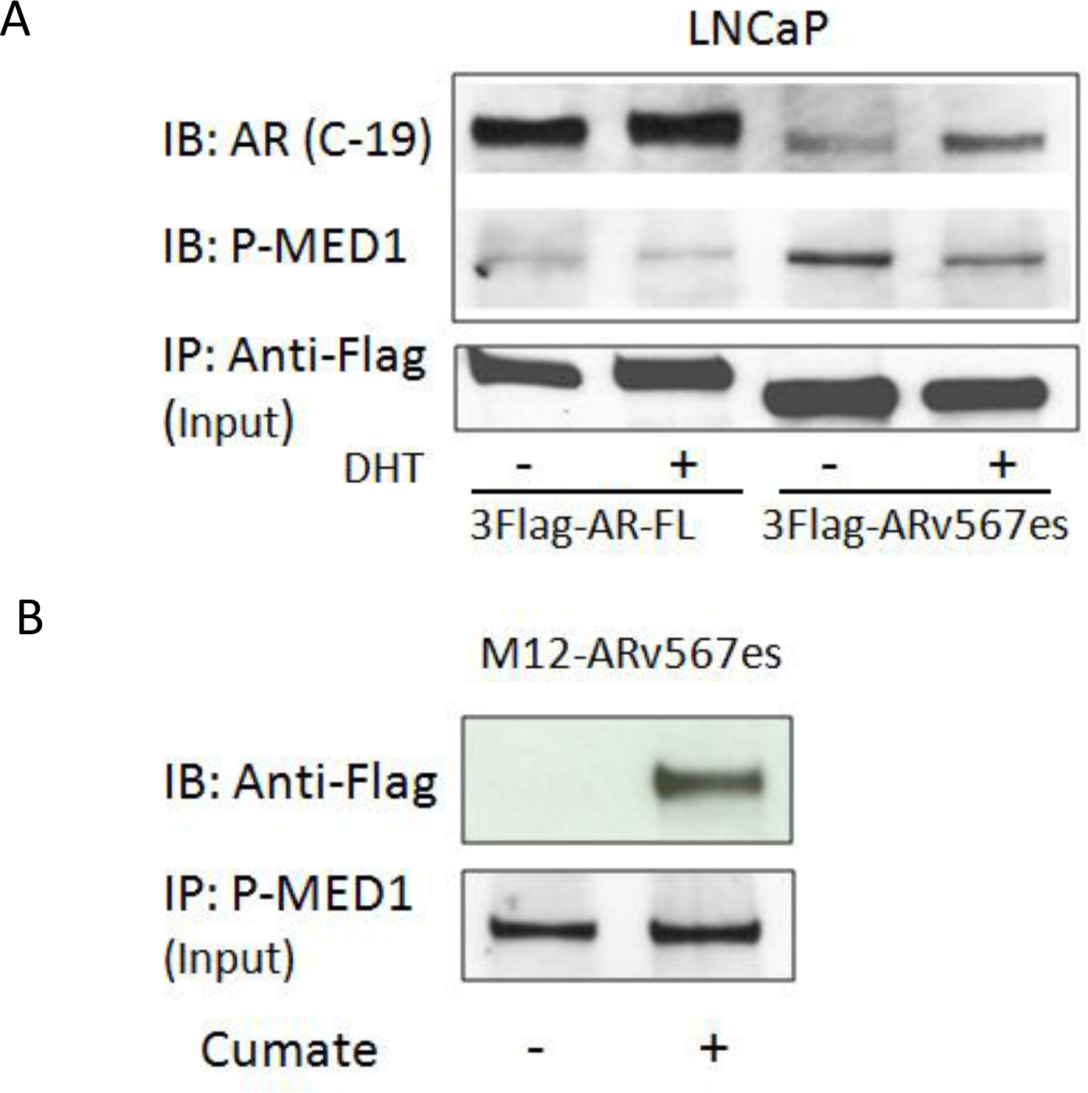
AR^v567es^ could bind p-MED1 independent of full length AR **(A)** In androgen-dependent LNCaP cells transiently transfected with Flag-tagged AR^v567es^, pulling-down of Flag could co-precipitate both AR^fl^ and p-MED1. With the same input volumes of 100ug, AR^v567es^ showed more abundant p-MED1 pull-down than that of full-length AR (AR^fl^). **(B)** Reversed pull-down with p-MED1 antibody could co-precipitate Flag-tagged AR^v567es^ in the cumate-inducible M12-ARv567es prostate cancer cell line.

As reported in a previous study we demonstrated that AR^v567es^ binds to full-length AR (AR^fl^ ) and increases the stability of AR^fl^ protein [19]. Here, we investigated whether there is a physical interaction between MED1 and AR^v567es^, and whether this interaction is mediated by full-length AR. The co-immunoprecipitation assay was performed with the LNCaP cell line transiently transfected with Flag-tagged AR^v567es^. As shown in Figure 2A, the anti-Flag antibody could pull down p-MED1 as well as AR^fl^, indicating a physical association of AR^v567es^ with p-MED1 and AR^fl^ in the context of protein activity. Of note, Flag-tagged AR^fl^ also pulled down p-MED1. However, with the same amount of input protein lysates (100 ug), AR^v567es^ showed abundance of p-MED1 co-precipitation especially in the absence of DHT, but AR^fl^ pulled down much less p-MED1 protein. This finding indicates AR^v567^ has more potency to recruit p-MED1 when androgen is depleted, which is exactly in accordance with the impaired cell growth by siMED1 in androgen-depleted condition shown in Figure 1C.

To rule out the possibility that AR^fl^ might mediate the interaction between AR^v567es^ and p-MED1, the AR-null M12 cell model was also used. As shown in Figure 2B, in the cumate inducible M12-AR^v567es^ stable cell line, the reverse pull-down of AR^v567es^ by p-MED1 antibody further demonstrated the physical interaction between these two molecules. The IgG controls showed the specificity of the co-IP binding (Fig. S4A, B). In total, these data indicate AR^v567es^, p-MED1, and AR^fl^ might form a ternary complex or bind separately, but AR^v567es^ bound to p-MED1 independent of full-length AR.

### The binding of phospho-MED1 to UBE2C promoter and enhancer increased when AR^v567es^ was present

**Figure 3:**
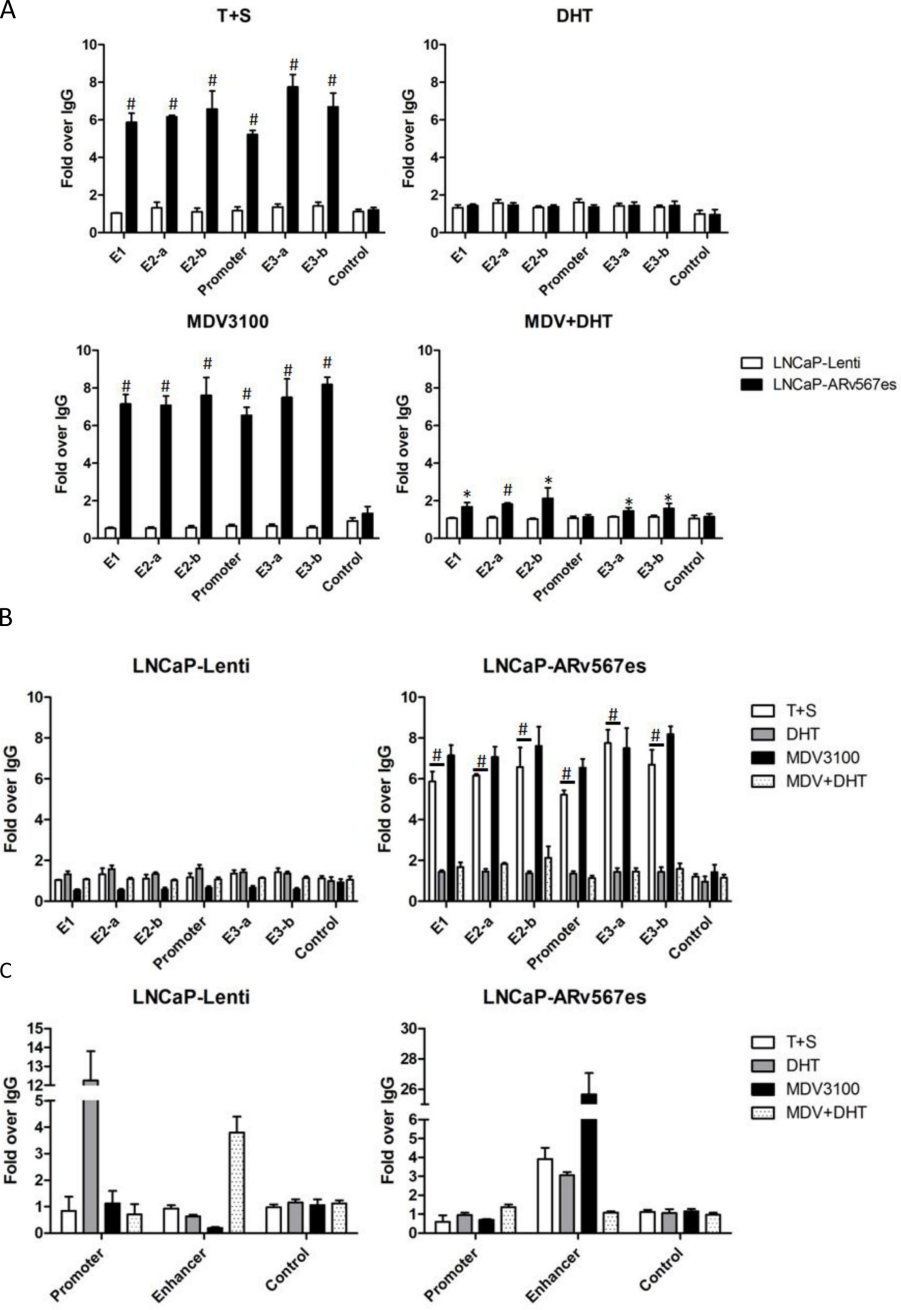
AR^v567es^ recruited p-MED1 to UBE2C enhancer and promoter regions independent of androgen **(A)** p-MED1 binding to UBE2C promoter and enhancers increased in LNCaP-AR^v567es^ cells compared with LNCaP-Lenti cells, in the conditions of androgen deprivation (T+S) and full-length AR inhibition (MDV, MDV+DHT); AR^fl^ activation by DHT abated the extra recruitment induced by AR^v567es^. **(B)** The reversed effect of DHT and MDV on the recruitment of p-MED1 to UBE2C promoter and enhancers in LNCaP-AR^v567es^ cells and LNCaP-Lenti cells. **(C)** AR^v567es^ also showed increased binding of p-MED1 to PSA enhancers but not in the promoter region especially when androgen receptor was inhibited (MDV3100). *p < 0.05 and #*p* < 0.01.

Since p-MED1 was recruited by AR^v567es^, we further tested whether AR^v567es^ could enhance p-MED1 binding to UBE2C transcriptional elements. As described by Chen et al, UBE2C promoter and enhancer regions can form a chromatin loop while triggering transcriptional initiation in CRPC cells [35]. Using ChIP assay, we tested p-MED1 binding capacity to the UBE2C promoter and all identified enhancers in LNCaP-Lenti and LNCaP-AR^v567es^ cells under different conditions. As shown in Figure 3A, when DHT was absent (T+S media), more p-MED1 binding (5–8 fold) occurred at UBE2C transcriptional regions, but not at control regions, in the LNCaP-AR^v567es^ cells compared to LNCaP-Lenti cells. Similar binding occurred when AR^fl^ was inhibited by enzalutamide (MDV3100), a potent AR LBD inhibitor [36]. However, in the presence of DHT, and thus AR^fl^ activation, the increased binding in LNCaP-AR^v567es^ cells diminished to a level seen in the LNCaP-Lenti cells. When both DHT and MDV3100 were present (MDV+DHT), a combination that partially inhibits AR^fl^ activity, the LNCaP-AR^v567es^ cell showed stronger p-MED1 binding than the LNCaP-Lenti cells, but still lower levels of binding than the LNCaP-AR^v567es^ T+S and MDV3100 treated groups (1–2 fold over IgG). These results strongly indicate that AR^v567es^ endows p-MED1 higher binding ability to UBE2C transcriptional regions in a ligand-independent manner. Consistent with the ChIP assay results, the UBE2C mRNA level (Fig. S5) exactly corresponded to the binding capacity of p-MED1 to UBE2C transcriptional elements. This indicates p-MED1 is the key mediator in ARv567es induced UBE2C transcription.

Figure 3A elucidates higher p-MED1 binding induced by AR^v567es^ than AR^fl^, and Figure 3B more clearly shows the divergent effects of androgen deprivation (T+S), AR^fl^ activation (DHT) and AR^fl^ inhibition (MDV3100) on p-MED1/UBE2C binding in these two cell lines. While androgen deprivation and AR^fl^ inhibition could abate p-MED1 binding to UBE2C in androgen-dependent LNCaP-Lenti cells, they could inversely and vigorously activate the p-MED1 binding to UBE2C in LNCaP-AR^v567es^ cells. This finding could coincidently address the mechanism on how androgen deprivation treatment (ADT) induces growth inhibition of androgen-dependent cancer, and also on how AR^v567es^ contributes to castration-resistance development. AR^fl^ activation (DHT) also has a contrary effect on these two cell lines. While DHT slightly strengthened the recruitment of p-MED1in LNCaP-Lenti cells, it significantly blocked this action in LNCaP-AR^v567es^ cells (*p* < 0.01). The negative regulation of DHT on AR^v567es^ activated p-MED1 binding was very consistent with our previous findings: DHT led to decreased cell proliferation (Fig. 1C) as well as lower amounts of p-MED1/AR^v567es^ precipitation (Fig. 2A).

In summary, these data lead to the following conclusions: 1) Transactivation of AR^fl^ by ligand confers low p-MED1 binding capacity to UBE2C transcriptional regions; 2) AR^v567es^ is a potent effector driving p-MED1 to the UBE2C locus independent of androgen; 3) When AR^fl^ and AR^v567es^ coexist and DHT is present, AR^fl^ takes priority, and recruits less p-MED1, thus facilitating low levels of binding to UBE2C transcriptional elements; 4) When androgens are depleted, AR^v567es^ takes the stage and recruits more p-MED1 resulting in much higher levels of binding to UBE2C regulatory regions. These findings are consistent with the chromatin-looping hypothesis [35] and could explain why the UBE2C promoter-enhancer loop is more likely to occur in CRPC cells but not in androgen-dependent prostate cancer cells.

As a control, PSA (KLK3) promoter and enhancer regions were also investigated with p-MED1 ChIP assays in LNCaP-AR^v567es^ cells (Fig. 3C). Very similar to UBE2C regulatory regions, p-MED1 showed high levels of binding to the PSA enhancer element when AR^fl^ was inhibited by MDV3100, and then next highest when DHT was absent (T+S). However, the p-MED1 binding to PSA promoter is not affected in all the treatment groups compared with IgG control, which is consistent with PSA mRNA outcomes (Fig. S5). These findings indicate that AR^v567es^/p-MED1 might perform alternative transcriptional regulation of AR canonical genes, but not necessarily affect the final gene expression.

### Phosphorylated MED1 mediates AR^v567es^ induced UBE2C locus transcription

**Figure 4:**
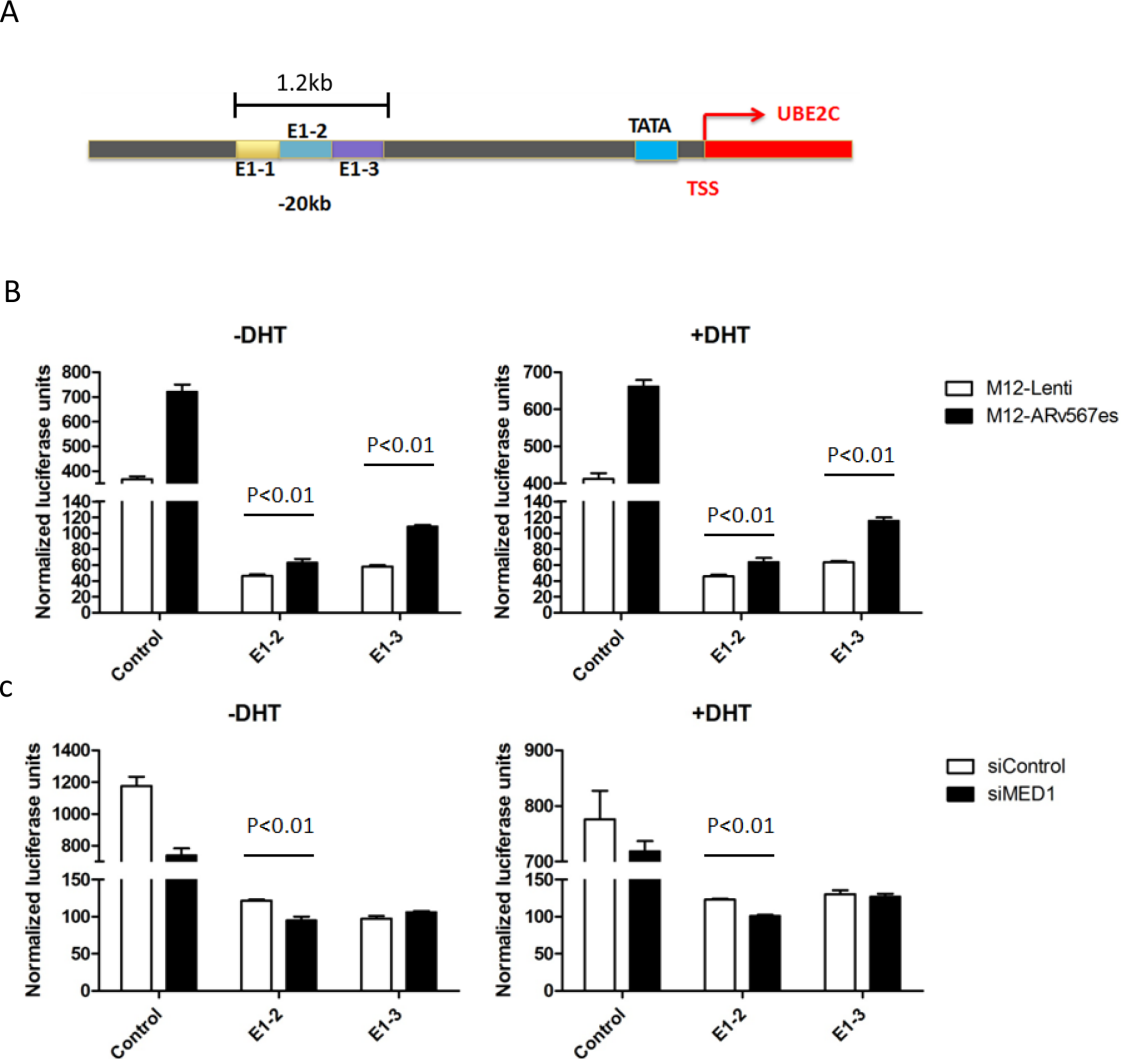
AR^v567es^ Increased UBE2C enhancer transcriptional activities **(A)** The diagrammatic view of the UBE2C Enhancer-1 region (20 kb upstream of the UBE2C transcriptional start site) used in the Luciferase reporter assay. **(B)** Even though lower than the E4TATA control, the luciferase activities of UBE2C E1-2 and E1-3 region were significantly higher ( *p* < 0.01) in M12-AR^v567es^ cell compared with LNCaP-Lenti cells. **(C)** MED1 silencing attenuated the transcriptional activation of AR^v567es^ on UBE2C enhancer specially showing significance on E1-2 ( *p* < 0.01).

We further studied the role of MED1 on AR^v567es^ induced transcriptional activity in the UBE2C locus. The 1.2 kb-long enhancer 1 (E1) fragment was used in a luciferase reporter assay (Fig. 4A). E1 is located 20 kb 5′ of the UBE2C transcription start site (TSS) and has the highest activity in a 3C assay [35]. Three ~400bp regions in Enhancer 1 termed E1-1, E1-2 and E1-3 were systematically subcloned into the pGL4.10-E4TATA-Luc vector. The reporter activities were measured in M12-Lenti cells and M12-AR^v567es^ cells. The M12 prostate cancer cell line is an AR negative line, so the effect of AR^fl^ could be excluded. While E1-1 showed comparable transcriptional activities in M12-Lenti and M12-AR^v567es^ cells (Fig. S6A, B), E1-2 and E1-3 both induced significantly higher Luc signals when AR^v567es^ was present ( *p* < 0.01) (Fig. 4B). After the expression of MED1 in M12-AR^v567es^ cells was silenced by siRNA, the Luc expression was inhibited subsequently (Fig. 4C). Similar results were seen in Chen et al's study, with control groups showing higher Luc activities in the LNCaP cell line, but much lower levels in PC3 cells. The reason might be due to very active E4TATA basal promoter activity in M12 and LNCaP cell lines. However, it does not affect these data, which indicated that AR^v567es^ enhanced UBE2C locus transcription and this enhancement was mediated by MED1.

### AR^v567es^/p-MED1 complex interaction with the PI3K/AKT pathway

**Figure 5:**
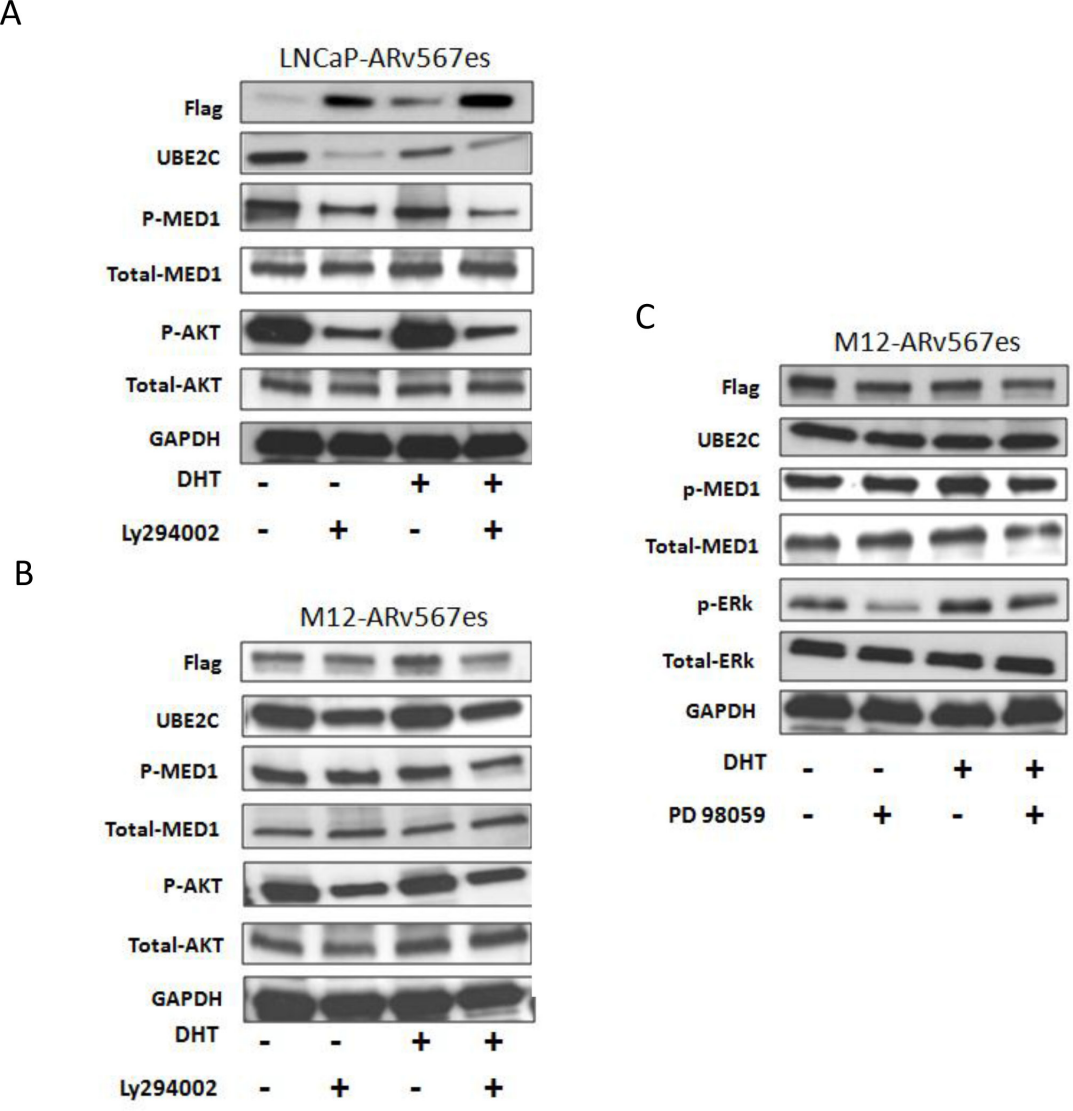
AR^v567es^/P-MED1 complex cross talks with PI3K-AKT pathway, but not with MAPK pathway Inhibition of PI3K kinase by LY294002 led to decreased MED1 phosphorylation and UBE2C expression both in LNCaP-AR^v567es^ cells **(A)** and M12-AR^v567es^ cells **(B)**. MAPK inhibitor PD98059 had no effect either on MED1 phosphorylation or UBE2C expression in M12-AR^v567es^ cells **(C)**.

The PTEN tumor suppressor gene is mutated in 50% of human prostate cancers. In addition, 70% of late stage prostate cancers show alterations in the PTEN/PI3K/AKT pathway [37]. We investigated whether the PI3K/AKT pathway is involved in AR^v567es^/p-MED1/UBE2C transcriptional activity. The PI3K inhibitor LY294002 was used to examine the effect of PI3K/AKT pathway inhibition on MED1 phosphorylation and UBE2C expression in LNCaP-AR^v567es^ and M12-AR^v567es^ cells. As shown in Figure 5(A, B), LY294002 reduced AKT phosphorylation at S473 and MED1 phosphorylation at T1457 leading to reduced UBE2C protein expression. The involvement of MAPK/ERK pathway was also investigated. Inhibition of the MAPK pathway by PD98059 did not have any effect on MED1 phosphorylation and UBE2C expression in M12-AR^v567es^ cells (Fig. 5C). p-ERK protein was non-detectable in LNCaP-AR^v567es^ cell by western blot, and UBE2C expression was not affected after treatment by PD98059 (data not shown). The involvement of PI3K/AKT, but not the MAPK pathway, in the proliferation of the LNCaP-AR^v567es^ cells was further observed in MTT assays by inhibited cell growth of LY294002 treated cells, but not of PD98059 (Fig. S7).

Another interesting finding we observed in this experiment was the decreased UBE2C protein expression in the LNCaP-AR^v567es^ cells when DHT was added to T+S media (Fig. 5A). This result not only addresses the direct mechanism of impaired LNCaP-AR^v567es^ cell growth under DHT stimulation (shown in Figure 1C), but also consistent with the negative regulatory effects of DHT on p-MED1 co-immunoprecipitation with AR^v567es^ (Figure 2A) and p-MED1 recruitment to the UBE2C locus (Fig. 3A, B).

### AR^v567es^/p-MED1 transcriptional regulation is associated with FoxA1

**Figure 6:**
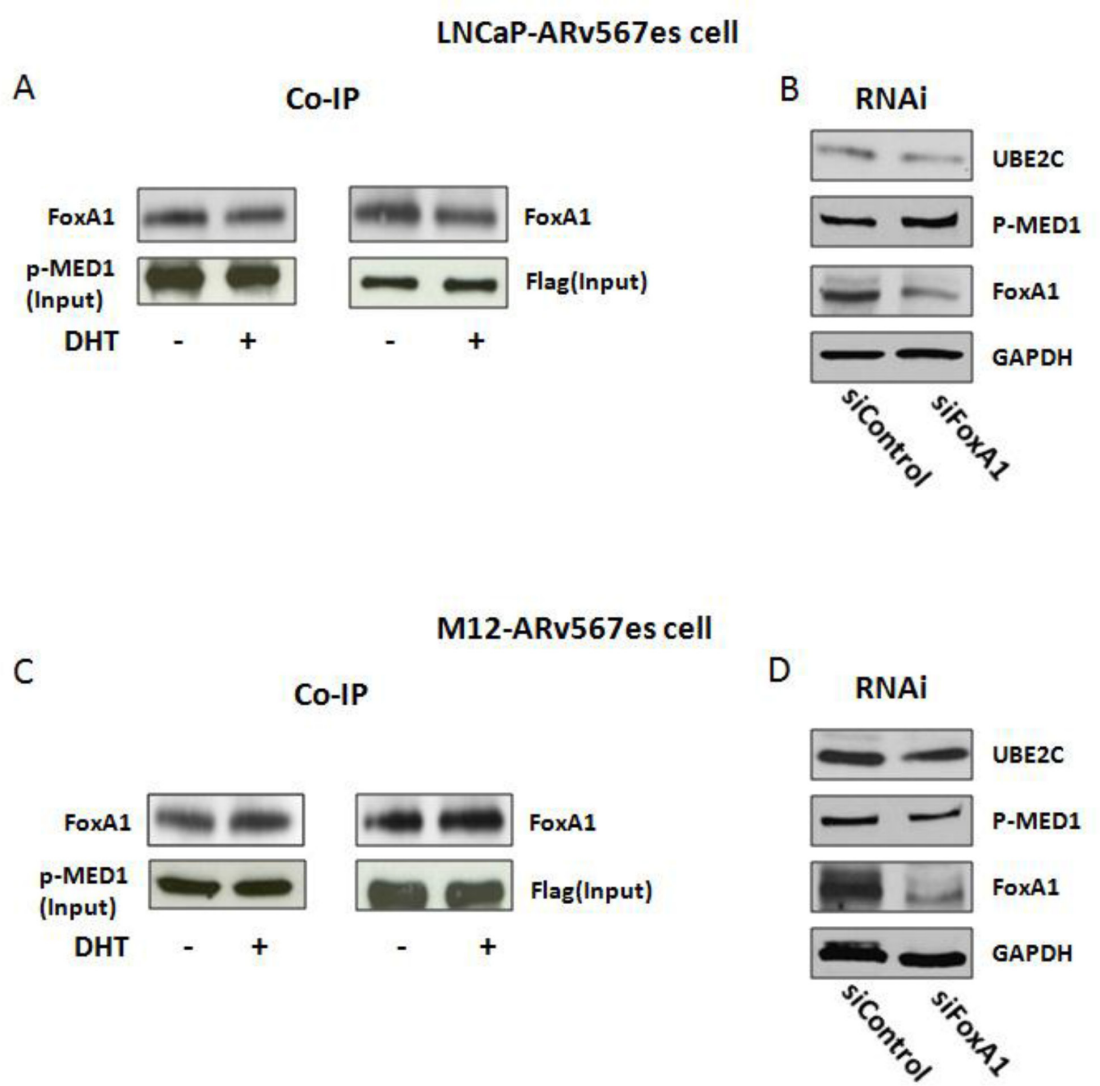
FoxA1 was involved in the AR^v567es^/p-MED1 transcriptional regulation **(A, C)** Co-IP showed the binding of FoxA1 to p-MED1 and AR^v567es^ in M12-AR^v567es^ and LNCaP–AR^v567es^ cell lines. **(B, D)** Silencing of FoxA1 decreased UBE2C expression in both cell lines.

As reported previously, phosphorylation of MED1 facilitates FoxA1, *Pol* II and TATA binding protein (TBP) recruitment and mediates their interactions on chromatin, leading to chromatin looping [35]. Here we performed co-IP with p-MED1 and Flag-tagged AR^v567es^ to examine the recruitment of FoxA1, a key component in the loop complex. In cumate inducible LNCaP-AR^v567es^ and M12-AR^v567es^ cells, p-MED1 antibody and Flag antibody were used as the pull-down antibodies followed by western blotting with the immune precipitated protein. As shown in Figure 6A and 6C, FoxA1 is co-precipitated with p-MED1 and Flag in both variant cell lines, which indicates FoxA1 is associated with the transcriptional complex of p-MED1/AR^v567es^, and this process is not dependent on AR^fl^. RNAi against FoxA1 demonstrated decreased expression of UBE2C in both cell lines (Fig. 6B, D), which further indicates the critical role of FoxA1 in AR^v567es^/p-MED1 transcriptional regulation. IgG controls were included in Figure S8 to show the specificity of the co-IP binding (Fig. S8A, B).

## DISCUSSION

The occurrence of ligand-independent, constitutively active androgen receptor splice variants in castration–resistant prostate cancer provides a conceptually simple explanation for the development of resistance to prostate cancer therapies that target the ligand-binding domain. The AR-Vs induce a distinct transcriptome in which overexpression of UBE2C and other cell cycle genes predominate, suggesting there might be different, or unique, transcriptional machinery used by AR variants compared to full-length AR.

MED1, also termed TRAP220, is a 220-kDa subunit of the human thyroid hormone receptor-associated protein (TRAP)-Mediator complex. TRAP Mediator has been described as a co-activator for a broad range of nuclear hormone receptors as well as other classes of transcriptional activators, including glucocorticoid receptor [38], estrogen receptor [39, 40] and androgen receptor [41]. Recently, the role of MED1 in prostate cancer oncogenesis and progression has gained recognition [33]. MED1 expression is upregulated in the epithelium of clinically localized human prostate cancer and in CRPC cell lines. Ectopic MED1 overexpression in prostate cancer xenografts significantly promoted tumor growth in nude mice [42]. Notably, MED1 overexpression in CRPC cells leads to upregulation of distinct AR target genes involving cell cycle progression, including UBE2C [23, 35]. Knockdown of MED1 resulted in cell-cycle arrest and decreased proliferation, which is also evident in our LNCaP-AR^v567es^ cell line.

It has been reported that phospho-MED1 mediates UBE2C locus looping in castration-resistant prostate cancer cells, but not in the androgen-dependent prostate cancer cells [35]. The chromatin looping model nicely elucidates the components in this complex, however it does not specify which transcription factor initiates looping formation given that MED1 interacts with multiple transcription factors as mentioned above. In the castrate-resistant cancer cells, there is low possibility for AR^fl^ still functioning actively with ligand depletion, but another story for constitutive active AR-Vs. Herein, we have identified that AR^v567es^ associates with p-MED1 as a key mediator in CRPC transcriptional activity. We found that: (i) MED1 is necessary for AR^v567es^ induced UBE2C up-regulation and subsequent prostate cancer cell growth; (ii) p-MED1 is recruited to AR^v567es^ independent of full-length AR; (iii) p-MED1 has higher recruitment to UBE2C promoter and enhancer regions in the presence of AR^v567es^, (iv) AR^v567es^ enhanced UBE2C transcription could be blocked by silencing MED1; (v) AR^v567es^/p-MED1 signaling crosstalks with the PI3K/AKT pathway but not the MAPK pathway, and (vi) FoxA1 is involved in AR^v567es^/p-MED1 induced UBE2C long range chromatin interactions.

Previous data demonstrated that an interaction between MED1 and AR worked in a ligand-dependent manner in androgen responsive prostate cells. However, a recent study showed MED1 could functionally interact with androgen receptor in a non-canonical way via a newly discovered binding motif in the AR N-terminal Tau-1 domain. [43] This study is of high interest to us, as the AR^v567es^ splice variant lacks the ligand-binding domain, its interaction with MED1 might be mediated by AR^fl^. However, based on this study we know that AR^v567es^, which contains the N-terminus, has the structural base to directly interact with MED1. Consistent with our data, we could see the binding of MED1 and AR^v567es^ through co-IP in AR positive LNCaP cells, but also in the AR-null M12 cells transfected with AR^v567es^. However, whether AR^v567es^ functionally interacts with MED1 through this particular Tau-1 domain needs to be further investigated.

**Figure 7:**
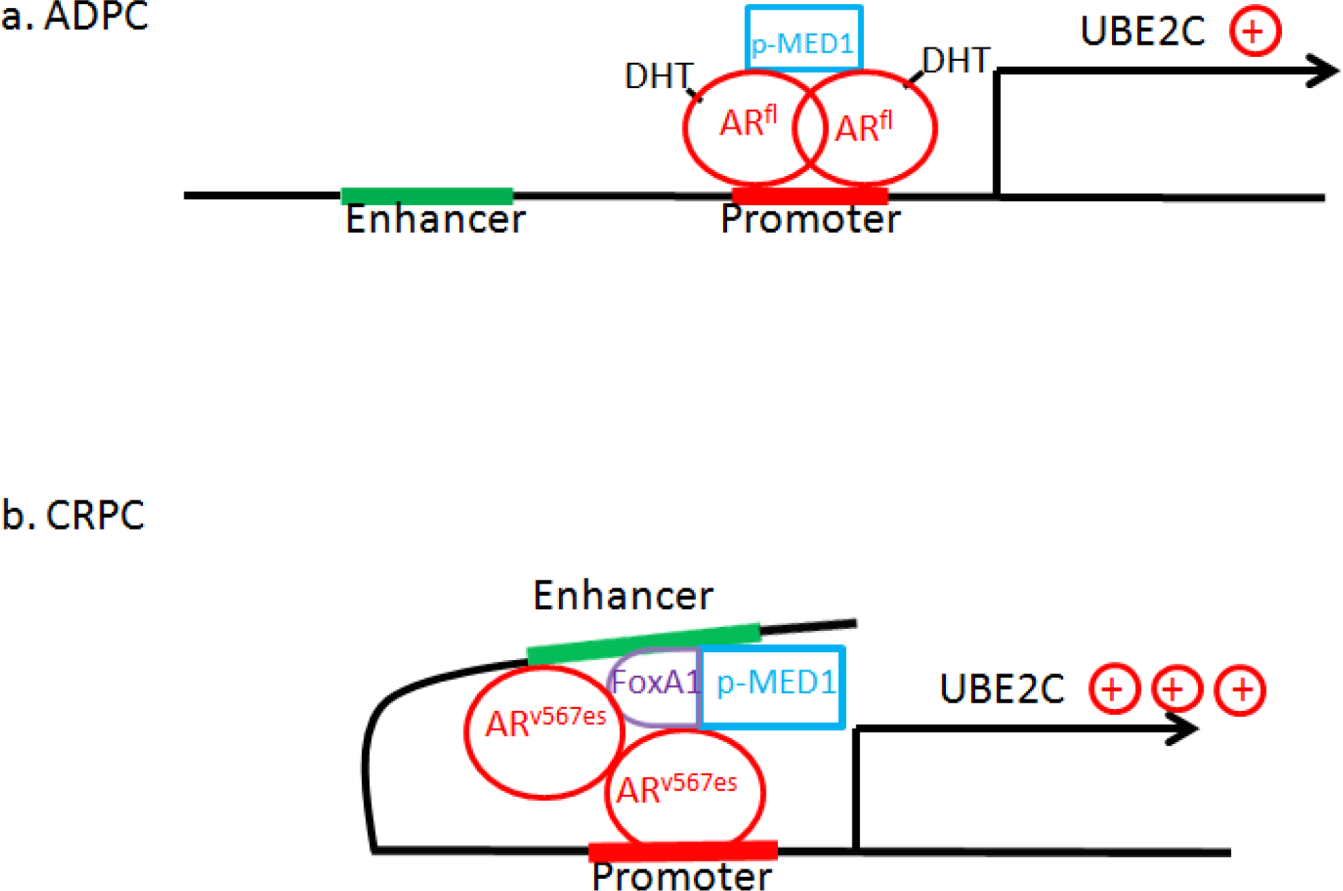
“The p-MED1 switch hypothesis”: Model of AR^fl^/AR^v567es^/p-MED1 transcriptional regulation on UBE2C in prostate cancer **(a)** In ADPC cells with the presence of DHT, p-MED1 goes to AR^fl^ but no chromatin looping forms and there is a low level of activation on UBE2C transcription; **(b)** In CRPC cells with DHT depleted, AR splice variants appear which have higher affinity to p-MED1, and recruit more p-MED1 to the UBE2C promoter and enhancers with the assistance of FoxA1, resulting in looping formation and vigorously activating UBE2C expression and leading to cell survival and increased cell proliferation.

The most significant finding in this study is the divergent mechanism of AR^fl^ and AR^v567es^ in regulating p-MED1 recruitment, driving p-MED1 to the UBE2C locus, and then affecting UBE2C expression and cancer cell growth. Here we raise a “p-MED1 switch” hypothesis (Fig. 7) that could reasonably address this: when androgen/DHT is available (prior to ADT) in androgen dependent prostate cancer (ADPC), more p-MED1 is recruited to AR^fl^ but no chromatin looping forms and AR^fl^ has a low level of activation on UBE2C transcription (Fig. 7A). After ADT therapy, the DHT is depleted and AR^fl^ signaling is inhibited, AR splice variants are formed as a survival mechanism due to the stress on the cells; the variants have higher affinity to p-MED1, and therefore recruit more p-MED1 to the UBE2C promoter and enhancers with the assistance of FoxA1, resulting in chromatin looping. This strongly enhances UBE2C expression, leading to the CRPC stage, which has enhanced cell survival and increased cell proliferation (Fig. 7B).

p-MED1 recruitment in castrate conditions to the UBE2C promoter/enhancer regions was very high in the presence of AR^v567es^. In the MDV+DHT treated LNCaP-AR^v567es^ cells, however, MDV3100 was not able to fully attenuate the DHT effect (Fig. 3B). The LNCaP-AR^v567es^ cells also express AR^fl^. MDV3100 has lower affinity to the AR^fl^ LBD and is not able to fully outcompete the ligand. Thus, the inability of MDV to overcome DHT's suppressive effect on p-MED1 recruitment in this cell line suggests that when DHT is present, it will facilitate the “p-MED1 switch” to AR^fl^ signaling leading to low levels of UBE2C transcription.

The recruitment of p-MED1 to AR canonical genes, such as PSA, differs from non-canonical genes, such as UBE2C. PSA ChIP in LNCaP-AR^v567es^ cells demonstrated that p-MED1 is only recruited to PSA enhancer regions, but not to the promoter. In contrast, in the LNCaP-Lenti cells p-MED1 had the strongest recruitment to the PSA promoter in the presence of DHT. PSA transcript levels overall are significantly lower in the LNCaP-AR^v567es^ cells compared with LNCaP-Lenti cells. Perhaps recruitment of p-MED1 to the enhancer regions of PSA is inhibitory whereas recruitment to the promoter region enhances transcription. These data indicate the “p-MED1 switch” hypothesis may not apply to canonical AR genes.

Multiple signaling pathways have been described leading to MED1 phosphorylation in a variety of conditions, including MAPK/ERK signaling [42] [44] [45, 46], AMP-activated protein kinase (AMPK) [47] and PI3K/AKT [35, 42]. In our study, we investigated ERK and AKT signaling pathways in LNCaP-AR^v567es^ cells and M12-AR^v567es^ cells, and found only the PI3K pathway was involved in phosphorylating MED1 in the context of AR variant. PTEN deletion with AKT pathway activation has long been recognized as one of the most important mechanisms of CRPC [48]. A previous study reported the activity of another AR variant, AR-V7, is regulated in a PTEN-PI3K-AKT-dependent way [49]; here we further confirmed the involvement of PI3K in AR-Vs' function.

The concurrence of the presence of constitutively active AR splice variant, increased MED1 and UBE2C, as well as crosstalk with the PI3K-AKT pathway, signifies that CRPC has multiple factors synergistically contributing to the process. Identification of potent inhibitors for AR-Vs and combining agents that target MED1, UBE2C and phospho-PI3K/AKT would provide an effective therapeutic strategy in future clinical trials.

## MATERIALS AND METHODS

### Cell culture, plasmid construction and transient transfection

LNCaP human prostate cancer cell line was obtained from the American Type Culture Collection. Early-passage cells were used in all experiments. These cells were grown in T-medium (Invitrogen, Grand Island NY, USA) supplemented with 10% FBS, 100 units/ml penicillin, and 100 ug/ml streptomycin at 37°C with 5% CO_2_. M12 cells are AR-negative and were obtained from Dr. J. Ware at the Medical College of Virginia. The generation and characterization of the M12 prostate cell line has been described previously [50–53]. M12 cells were cultured in RPMI 1640 medium (Invitrogen) containing 5% FBS, 10 ng/ml EGF, 0.02 mM dexamethasone, 5 ug/ml insulin, 5 ug/ml transfection, 5 ng/ml selenium, fungizone, and gentamicin at 37°C with 5% CO_2_. cDNA of the entire AR^v567es^ variant and AR^fl^ were cloned into p3xFlag-CMV-9 vector as described previously [19, 53]. The expression constructs were transfected into the human prostate cancer cell lines using TurboFect reagent according to the manufacturer's protocol (Thermo Scientific, Pittsburgh PA, USA).

### Generation of AR^v567es^ expressing stable cell lines

LNCaP and M12 cell lines expressing cumate-inducible 3xflag tagged AR^v567es^, LNCaP-AR^v567es^ and M12-AR^v567es^, were made using the SparQ™ cumate switch lentivector system (Systems Biosciences, Mountain View, CA). Briefly, the flag-tagged AR^v567es^ sequence was first subcloned into the lentiviral expression vector pCDH-EF1-CymR-T2A-Puro and then packaged into lentiviral particles using pPACK™ packaging systems (System Biosciences). Finally, LNCaP and M12 cells were infected with 1 × 10^7^ virus particles per 1 × 10^6^ cells then selected with 1 ug/ml puromycin (Invitrogen) for 10 days. Cells infected by empty lentivectors, LNCaP-Lenti/M12-Lenti, were used as control cells.

### Quantitative real-time PCR

Total RNA was isolated using Trizol reagent according to the manufacturer's instructions (Invitrogen). cDNA was reverse transcribed from total RNA (1 ug) using iScript cDNA Synthesis Kit (Bio-Rad, Hercules, CA). Real-time polymerase chain reactions were performed using SYBR Green PCR Master Mix (Applied Biosystems, Grand Island, NY) on a ViiA 7 Real Time-PCR System (Applied Biosystems) following the manufacturer's instructions. Primer sequences and Taqman probe sequences used in this study are listed in Table 1. The housekeeping gene RPL13A was used as an endogenous control.

**Table 1:** QPCR Primer sequences

Target gene	Primer Sequence (5′to 3′)
UBE2C+	TGGTCTGCCCTGTATGATGT
UBE2C-	AAAAGCTGTGGGGTTTTTCC
PSA+	CAACCTGCAAACCTAGGGAA
PSA-	TCAGGGTTGACAGGAGGAAC
RPL13A+	CCTGGAGGAGAAGAGGAAAGAGA
RPL13A-	TTGAGGACCTCTGTGTATTTGTCAA

### Proliferation assay

100 ul of cells were seeded at the concentration of 10,000 per well in 96-well plates in serum free media containing transferrin and selenium (T+S). For cells with dihydrogestosterone (DHT) treatment, 10^−9^ M was added. Cells were allowed to proliferate for 24, 48, 72, and 96 hrs respectively. 20 ul/well of CellTiter 96 AQueous One Solution reagent was added and incubated at 37°C for two hours prior to reading (Promega, Madison WI, USA). The absorbance was then recorded using iMark Microplate Reader (Bio-Rad) at a wavelength of 490 nm. Each point represents the mean ± SD of 3 replicates.

### RNA interference (RNAi)

Small interfering RNA (siRNA) duplexes for MED1 (siMED1) and FoxA1 (siFoxA1), as well as scrambled negative controls (siControl) were purchased from OriGene (Rockville, MD). The siRNA sequences are listed in Table 2. siRNA duplexes were transfected using TurboFect reagent according to the manufacturer's instructions (Thermo Scientific).

**Table 2:** siRNA sequences

siMED1	(1)GGAUUAGACAGCAAACCAGGGAAGC (2)AGCUGUAAACUCUACAAAGGGCUGT (3)AGAUGUCAGUAUACGAAACAUUATT
siFoxA1	(1)GGAGGAGAGAUAAGUUAUAGGGAGC (2)CUCUUAACCAUAAGAAUUGAAAUGG (3)GAAGUUUAAUGAUCCACAAGUGUAT

### Western blotting

Cells were washed with PBS and lysed with cold lysis buffer (50 Mm HEPES, 150 mM NaCl, 1.5 mM EGTA, 1% Triton X-100) containing protease inhibitors (Complete Mini Tablets) (Roche, USA) and Phosphatase Inhibitors Cocktail II (Sigma-Aldrich, USA). Protein concentration of cell extracts was determined by the BCA Protein assay (Thermo Scientific). Twenty micrograms of protein was electrophoresed through 4–15% gradient SDS-PAGE and subsequently transferred onto nitrocellulose membranes using Invitrogen iBlot Gel transfer system, and probed with respective antibodies at 4°C overnight. Antibodies used in this study and the working conditions are listed in Table 3. The blots were washed and incubated with a horseradish peroxidase-conjugated secondary antibodies (Pharmacia Biotech, Piscataway, NJ) for 1 hour at room temperature. Immunoreactive proteins were detected by ECL (Pharmacia Biotech). The membranes were stripped for 30 minutes in stripping buffer (Pierce, Rockford, IL) and reprobed with anti-GAPDH antibody as a loading control.

**Table 3:** Antibodies and reagents

1^st^/2^nd^Antibody/Reagent	Company
Anti-Flag	Sigma
Anti-AR (C-19)	Santa Cruz
Anti-AR (N20)	Santa Cruz
Anti-MED1	Santa Cruz
Anti-p-MED1	Abcam
Anti-UBE2C	Boston Biochem
Anti-AKT	Cell Signaling
Anti-p-AKT	Cell Signaling
Anti-ERK	Cell Signaling
Anti-p-ERK	Cell Signaling
Anti-H3K4ME1	Abcam
Anti-H3K4ME3	Abcam
Anti-FoxA1	Thermo Scientific
Anti-AR-V7	Precision Antibody
GADPH	Cell Signaling
Anti-Rabbit IgG	Pharmacia Biotech
Anti-Mouse IgG	Pharmacia Biotech
Dihydrotestosterone (DHT)	Sigma
MDV3100 (Enzalutamide)	Selleck Chemicals
LY294002	Sigma
PD98059	Cell Signaling

### Co-Immunoprecipitation

Cells were lysed in cold lysis buffer using the aforementioned lysis buffer with complete protease and phosphatase inhibitors (Roche Applied Science). Precleared cell lysate was incubated with anti-Flag or anti-p-MED1 antibodies overnight and then with ultralink immobilized protein A/G plus beads (Thermo Scientific) for 24 hours. Beads were washed four times with cold lysis buffer, and then samples were degenerated by boiling. Lastly, immune complexes were applied in Western blotting as previously described.

### ChIP assay

ChIP assays were performed using MAGnigy^TM^ Chromatin Immunoprecipitation System (Invitrogen) according to the manufacturer's instructions. Briefly, chromatin was crosslinked for 10 min at room temperature with 1% formaldehyde. After sonication with Diagenode Bioruptor (Denville, NJ), chromatin was sheared into fragments of ~500bp and immunoprecipitated with Dynabeads coupled with anti-p-MED1 antibody or isotope control. After washing, the reversed ChIP DNA was purified and then analyzed by real-time PCR. The primers used are listed in Table 4.

**Table 4:** ChIP Primer sequences

**UBE2C**	
E1+	TGCCATGTGCCCTAGAAACTG
E1−	CAAGCTCAGCAAAATGGTGAAA
Promoter +	GCCCGAGGGAAATTGGAT
Promoter −	TTACTCCGCGTGGGAACACT
E2-a+	GTGCGTGGTGGATCAAGTTATC
E2-a−	GGGTGCTCATCCCCATGA
E2-b+	TCCTTTTTAGGGACATGGATGAAG
E2-b−	GTGTTTGGTTTTTTGTCCTTGTGAT
E3-a+	CCCTGGTGGGCCTAGATGA
E3-a−	CAACTTCTCCCTTCCCCTGTCT
E3-b+	CATCCCCCCACACGAAGTTA
E3-b−	TGGATAGGGAGGGTCTTGTATGA
**UBE2C**	
Control +	CCACAAACTCTTCTCAGCTGGG
Control −	TTCTTTCCTTCCCTGTTACCCC
**PSA**	
Enhancer +	GCCTGGATCTGAGAGAGATATCATC
Enhancer −	ACACCTTTTTTTTTCTGGATTGTTG
Promoter +	CCTAGATGAAGTCTCCATGAGCTACA
Promoter −	GGAGGGAGAGCTAGCATTG

### Luciferase reporter assay

The pGL4.10-E4TATA-Luc vectors with UBE2C Enhancer-1 (E-1) sequences and pBEC22 (a Renilla luciferase vector) were kindly provided by Dr. Qianben Wang [35]. Three ~400 bp regions in Enhancer 1 called E1-1, E1-2 and E1-3 were systematically subcloned into pGL4.10-E4TATA-Luc vector. pGL4.10-E4TATA-Luc and pBEC22 were created by insertion of a 50-bp minimal E4 TATA promoter sequence, driving luciferase expression, into Bgl II to Hind III sites of vectors pGL4.10 and pGL4.70 (Promega) [54, 55]. For reporter assays, transfection was performed using Turbofect transfection reagent (Thermo Scientific). Cells were allowed to grow for another 24 hr before harvest. Luciferase activity was measured using a Dual-Luciferase Reporter Assay kit and GloMaxTM System (Promega).

### Statistical analyses

All the experiments were performed at least three times. The data are displayed as mean ± SEM. When two groups were compared, 2-tailed Student's t test was used (GraphPad Prism, version5.0d; GraphPad Software, La Jolla, CA). A *p* value of 0.05 or less was considered significant.

## SUPPLEMENTARY FIGURES


