# Supplementary material for: MED1 mediates androgen receptor splice variant induced gene expression in the absence of ligand
Source: Oncotarget. 2014 Dec 3;6(1):288–304. doi: 10.18632/oncotarget.2672 (PMC4381595; doi:10.18632/oncotarget.2672)
Supplement: Supplementary file 1 [file oncotarget-06-288-s001.pdf]

## SUPPLEMENTARY FIGURES

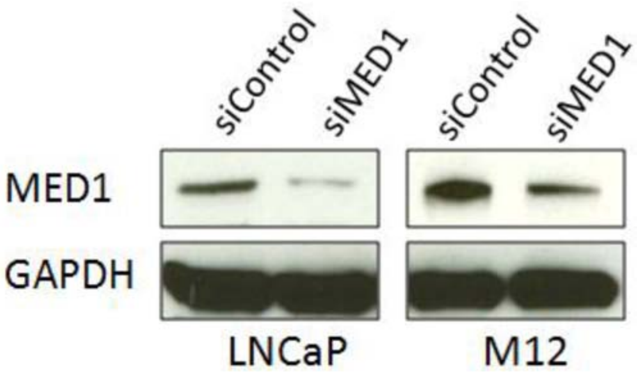


**Supplementary Figure S1: Suppression of MED1 levels by siRNAs.**

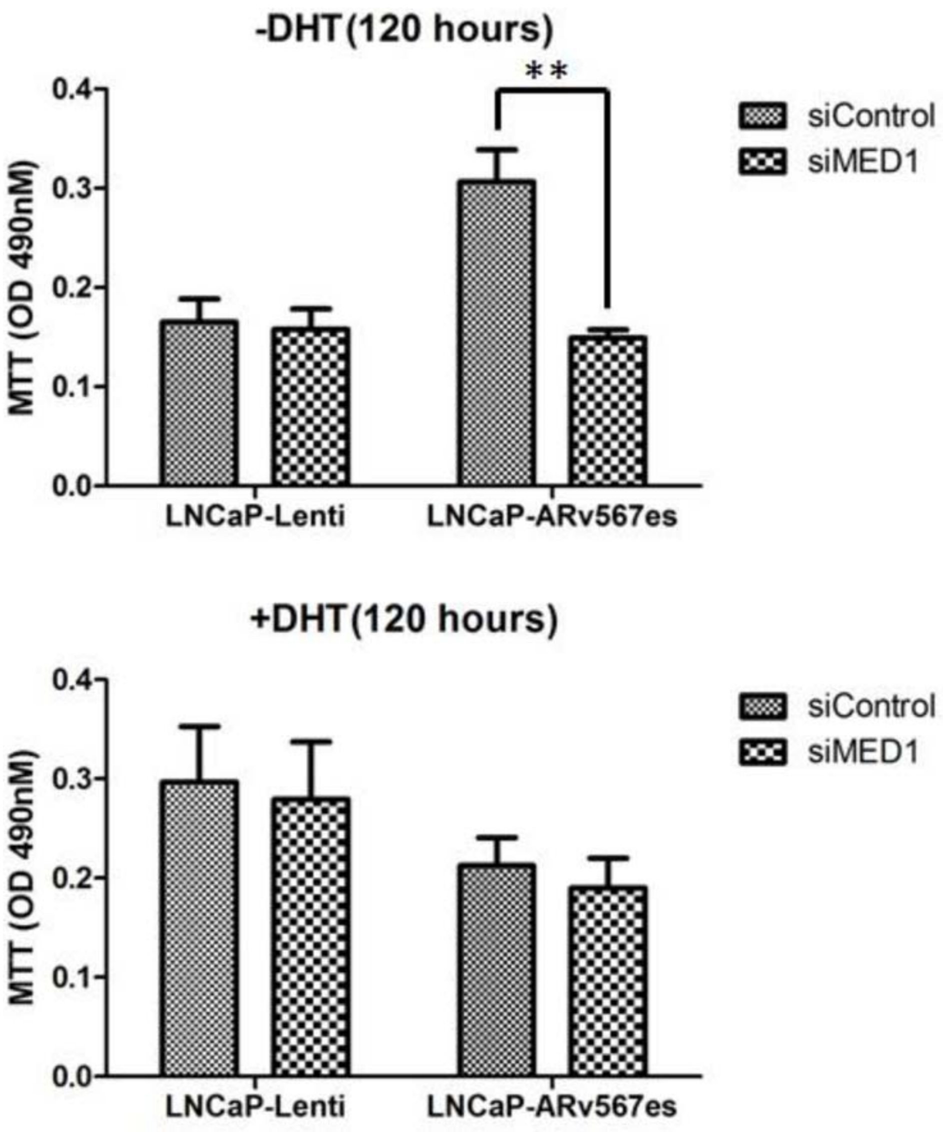


**Supplementary Figure S2: 120-hour MTT assay with the LNCaP-Lenti and LNCaP-ARv567es cells treated with siMED1 RNA duplexes or scramble controls under androgen-deprived condition or DHT.** siMED1 only showed significant growth inhibition (***$p < 0.01$*) of the LNCaP-ARv567es cells in the absence of DHT. These data are complimentary to Figure 1C. Values are mean ± SEM.

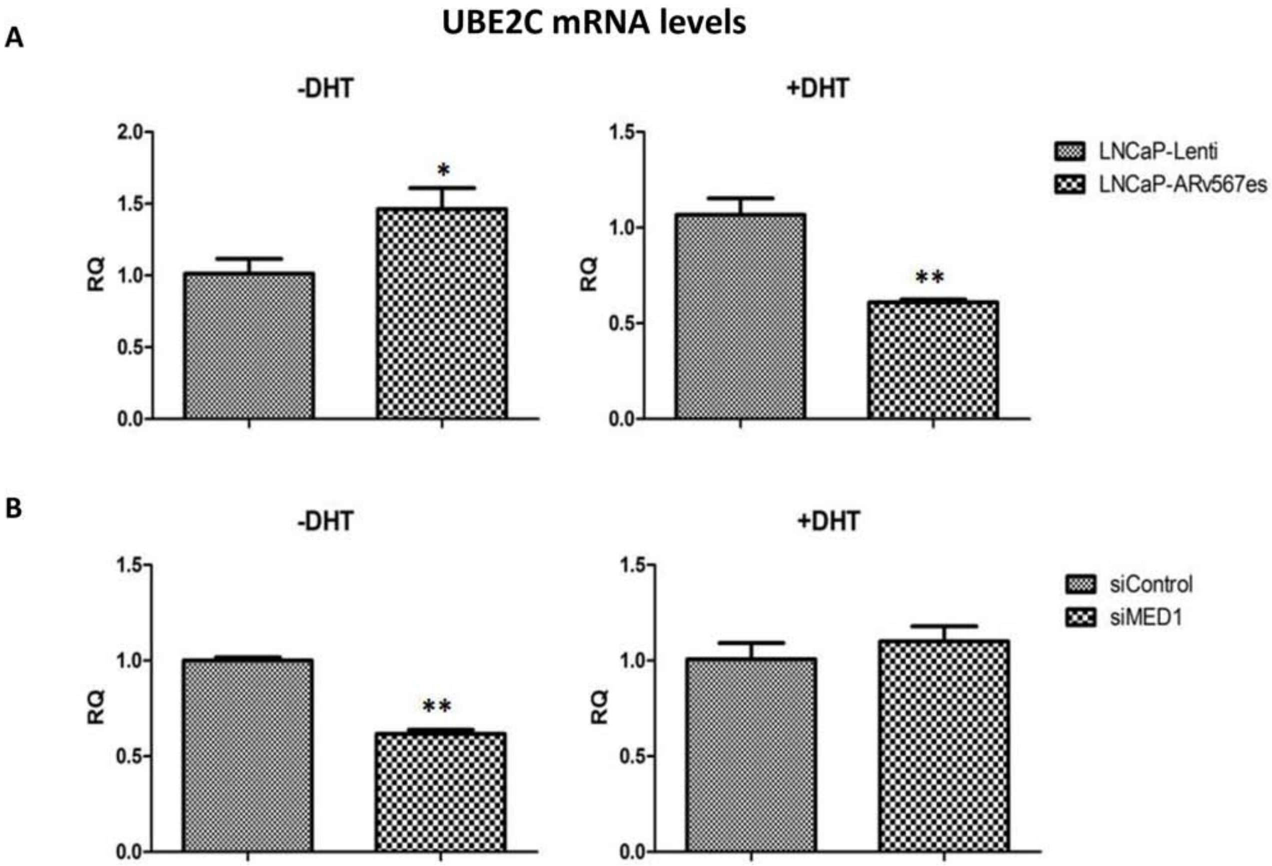


**Supplementary Figure S3: UBE2C expression in LNCaP –ARv567es stable cell line. (A)** Increased UBE2C mRNA level in androgen-deprived (-DHT) LNCaP-ARv567es cell line could be reversed by addition of DHT ($10^{-9}$M). **(B)** In LNCaP-ARv567es cell line, MED1 silencing could inhibit UBE2C transcription only when DHT was absent (-DHT), but not in the presence of androgen. Values are mean ± SEM. *$*p < 0.05$ and $**p < 0.01$*.

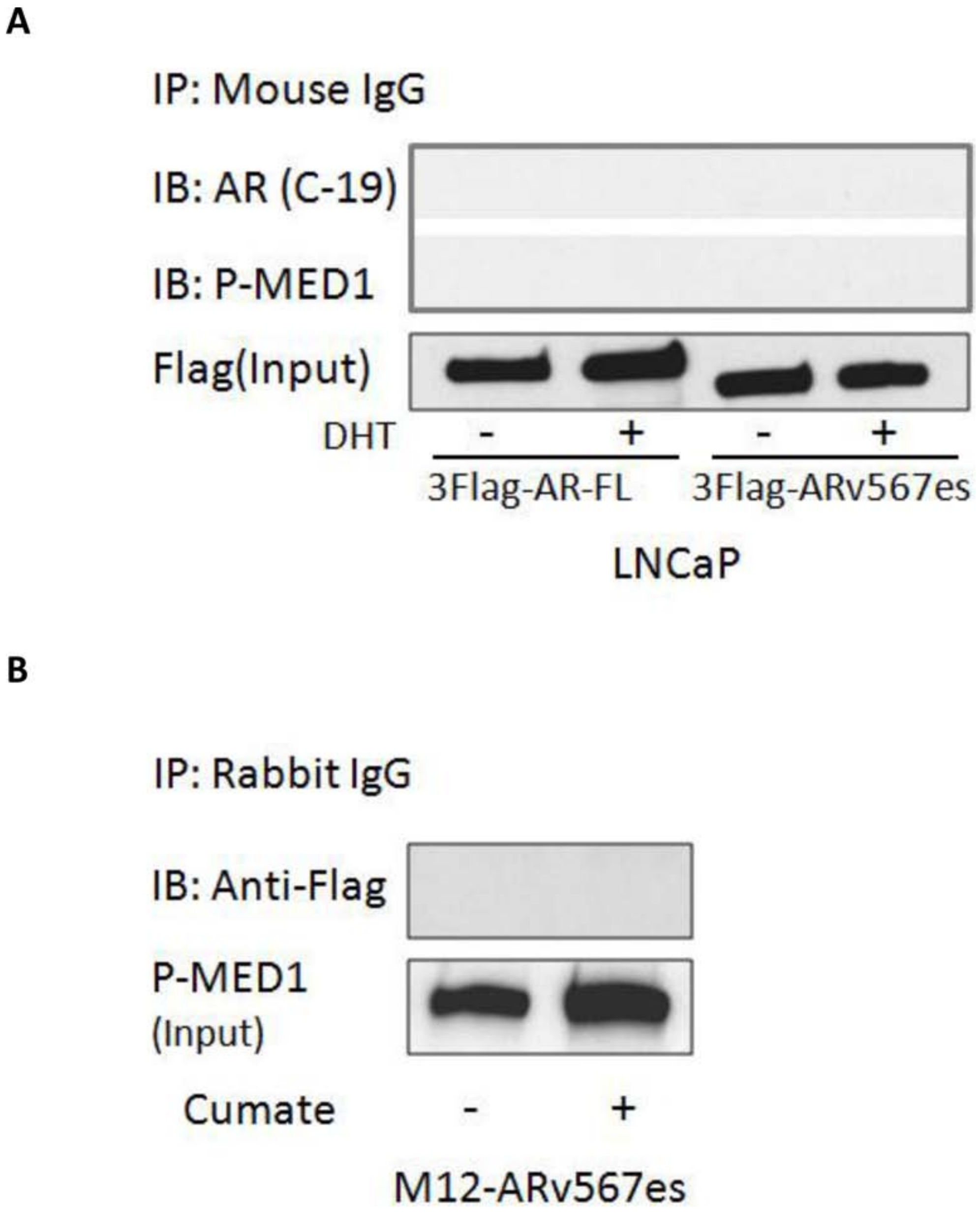


**Supplementary Figure S4: Mouse IgG control for Flag co-IP in LNCaP cells transiently transfected with 3Flag-AR-FL and 3Flag-ARv567es vectors (A); Rabbit IgG control for p-MED1 co-IP in M12-ARv567es cells (B).**

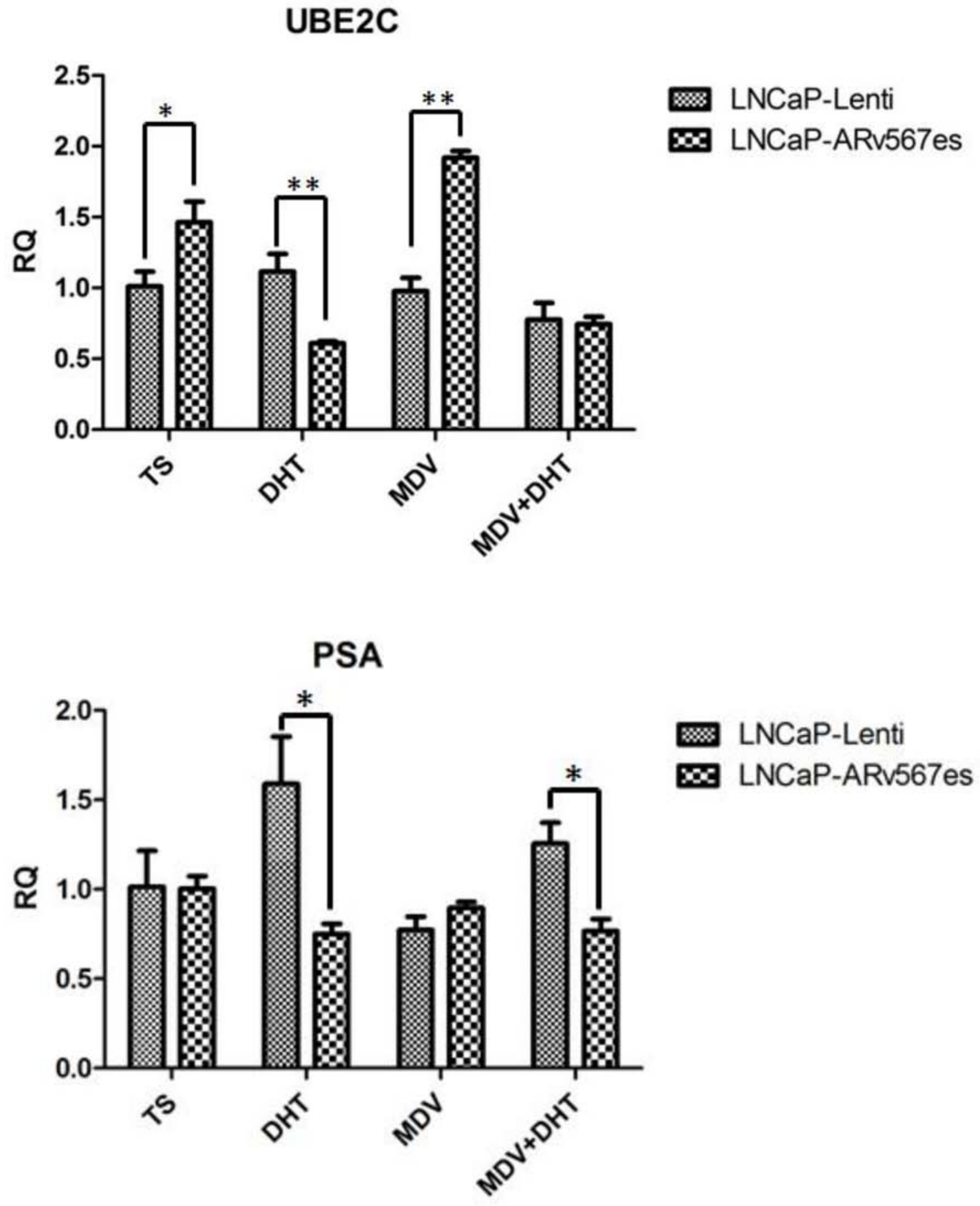


**Supplementary Figure S5: The mRNA level of UBE2C and PSA in LNCaP-Lenti and LNCaP-ARv567es cell line in androgen-deprived condition (TS), DHT (1nM), MDV3100 (10uM) and MDV+DHT.** Values are mean ± SEM. *$p < 0.05$ and **$p < 0.01$. These data are complimentary to the ChIP assay of Figure 3.

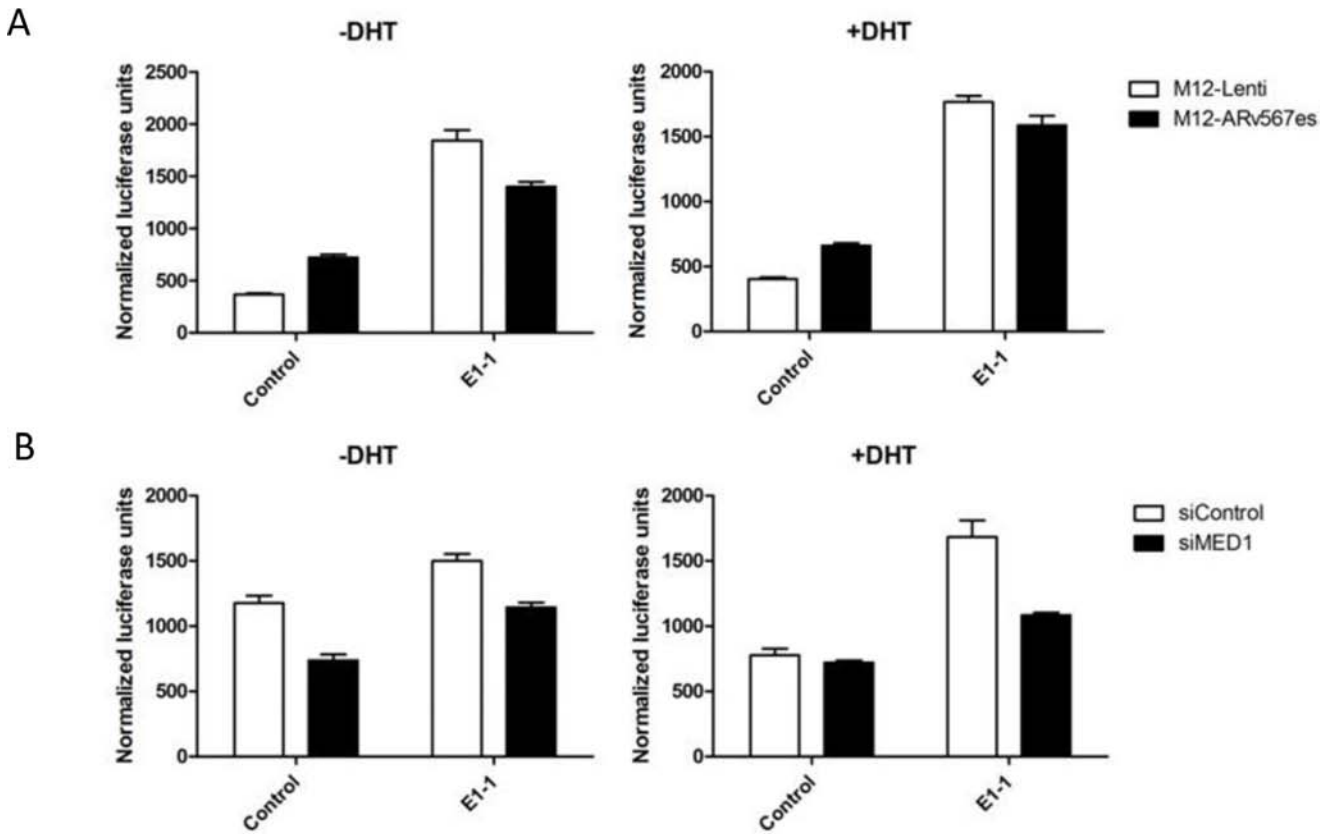


**Supplementary Figure S6: (A) UBE2C enhancer E1-1 has comparable transcriptional activity in M12-Lenti and M12 –ARv567es cell lines; (B) The activity in M12-ARv567es cell line could be suppressed after MED1 silencing.**

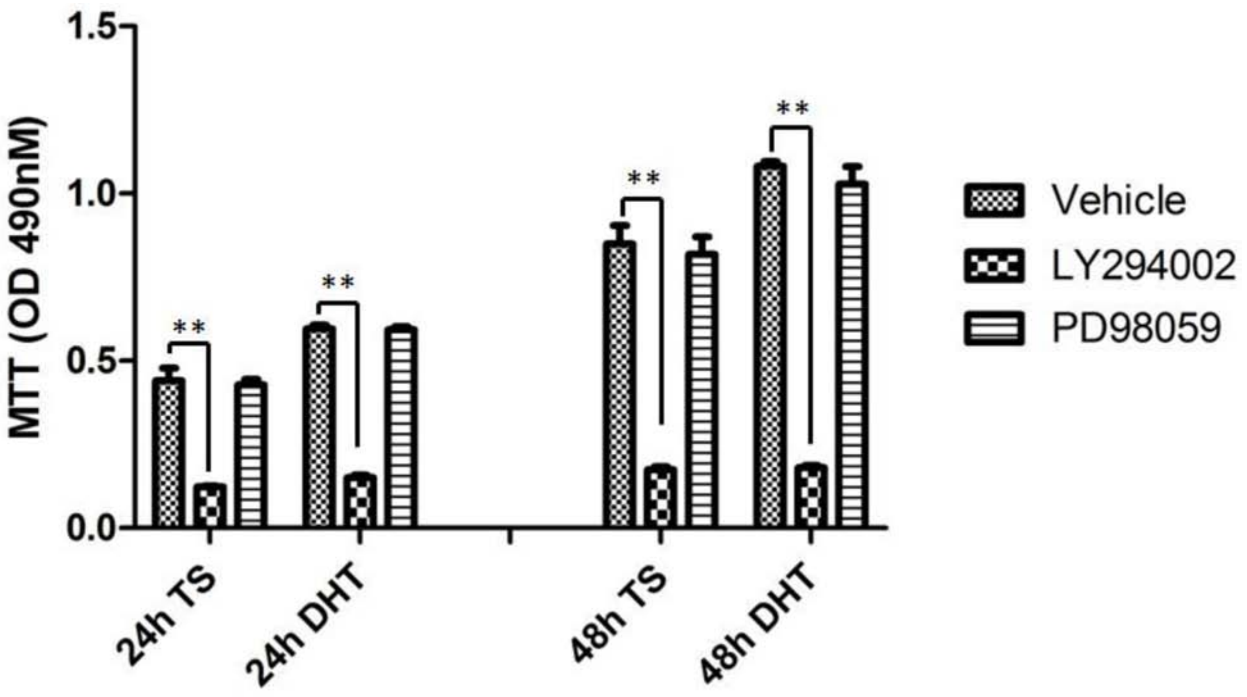


**Supplementary Figure S7: Cell proliferation assay (MTT) of LNCaP-AR$^{v567es}$ cells treated by PI3K inhibitor LY294002 (25 uM), and MAPK inhibitor PD98059 (10 uM) for 24 and 48 hours.** **$p < 0.01$

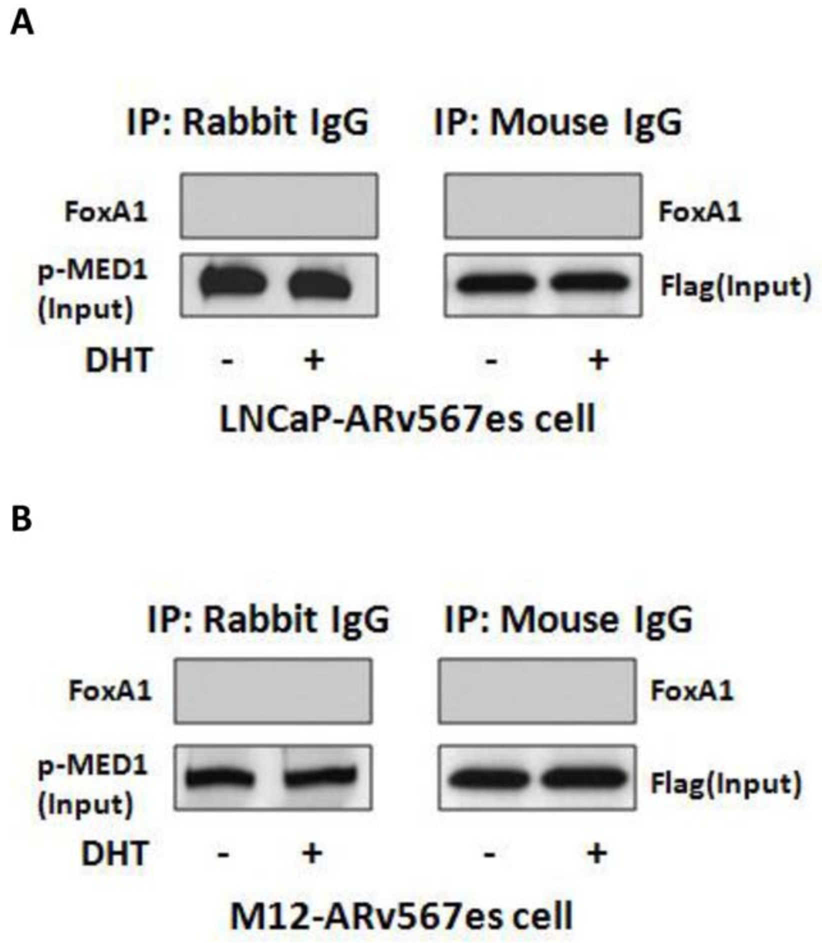


**Supplementary Figure S8: IgG controls for p-MED1 and Flag co-IP in LNCaP-ARv567es (A) and M12-AR$^{v567es}$ cells (B).**
